# The posterior cortical axis as an alternative reference for femoral component placement in total knee arthroplasty

**DOI:** 10.1186/s13018-020-02146-y

**Published:** 2020-12-11

**Authors:** Ji-Hoon Nam, Yong-Gon Koh, Kiwon Kang, Joon-Hee Park, Kyoung-Tak Kang

**Affiliations:** 1 Department of Mechanical Engineering, Yonsei University, 50 Yonsei-ro, Seodaemun-gu, Seoul, 03722 Republic of Korea; 2 Joint Reconstruction Center, Department of Orthopaedic Surgery, Yonsei Sarang Hospital, 10 Hyoryeong-ro, Seocho-gu, Seoul, 06698 Republic of Korea; 3 Orthopaedic Clinic, Gaja Yonsei Hospital, A-304, 7, Janggogae-ro 337 beon-gil, Seo-gu, Incheon, Republic of Korea; 4 Department of Anesthesiology & Pain Medicine, Hallym University College of Medicine and Kangdong Sacred Heart Hospital, 150 Seongan-ro, Gangdong-gu, Seoul, 05355 Republic of Korea

**Keywords:** South Korean patients, Trochlear anterior line, Femoral posterior cortical axis, Morphometry, Total knee replacement

## Abstract

**Background:**

Although several reference axes have been established for determining femoral rotational alignment during total knee arthroplasty (TKA), the most accurate axis is undetermined. This study determines the relationship between the posterior cortical axis (PCA) and the trochlear anterior line (TAL) of the femur in relation to the epicondylar axis.

**Methods:**

A total of 341 patients who underwent TKA for osteoarthritis were enrolled. Patients who had undergone previous bony surgery or replacement that might have changed the femoral geometry were excluded. Finally, 336 patients (200 females and 136 males) were included in the study. The angles between the transepicondylar axis (TEA) and TAL and TEA and the femoral PCA (FPCA) were evaluated. We also assessed whether there was any significant differences in variance and gender in these two angles. Student’s *t* tests were used to determine the significance of coronal alignment and any gender-based differences. The variances between the TAL/TEA and FPCA/TEA angles were compared using *F* tests.

**Results:**

The FPCA was externally rotated by 2.6° ± 3.6°, and the trochlear anterior line was internally rotated by 5.2° ± 5.5°, relative to the TEA. Gender-based differences were observed in the comparisons between anatomical references and TEA.

**Conclusions:**

The FPCA is a more conservative landmark than the TAL for intraoperative or postoperative approximation of the TEA. When conventional reference axes, such as the posterior condylar axis and the anteroposterior axis, are inaccurate, surgeons can refer to this alternative reference. These findings demonstrate that the FPCA may be useful for determining the rotational alignment of the femoral component before and during TKA.

## Introduction

The femoral component alignment of internal-external rotation in total knee arthroplasty (TKA) can influence the long-term clinical results [1, 2]. Improper alignment can cause tibiofemoral joint instability or loosening of the tibial component and patellofemoral maltracking associated with subluxation or dislocation of the patella [3, 4]. Most studies have used three axes defined by bony landmarks to guide the femoral component rotational alignment the transepicondylar axis (TEA) [5], the posterior condylar axis, and Whiteside’s line [6]. Cadaver and radiological studies have reported that the TEA is parallel to the flexion-extension axis of the knee, as reported in [7, 8]. However, manual palpation of the TEA is unreliable during surgery and is not reproducible as the anatomical reference point used for identifying the TEA is covered with soft tissue [9]. It is difficult to identify the sulcus of the medial epicondyle by palpation, and variations in the shapes of the epicondyles make it difficult for surgeons to recognize the TEA reference points [10]. During the postoperative evaluation of component rotation using computed tomography (CT), Whiteside’s line and the posterior condylar axis are unavailable as references; hence, the TEA must be determined directly [11]. However, femoral implants, particularly in implants with femoral boxes, may restrict the determination of TEA [11].

Therefore, recent studies have suggested two reference axes in the anterior and posterior femur as alternatives to the conventional reference axes in the event of distortion. The trochlear anterior line (TAL) is a line connecting the most anterior points of the medial and lateral femoral condyles [12–15], whereas the femoral posterior cortical axis (FPCA) is a line parallel to the posterior surface, proximal to the point where the femoral trochlea ends [11]. These studies have provided not only valuable supplementary information for the rotational alignment of the femoral component but also important anthropometric data of the distal femur that can be used for designing implant.

Despite these potential benefits, no studies have compared these two new reference axes.

Therefore, this study evaluated the relationship between the TAL and the FPCA to elucidate the reliability of each reference axis by comparing their variances. We used magnetic resonance (MR) images of knee joints with osteoarthritis of 336 South Korean patients (220 female and 136 male). We also investigated gender-based differences in the measurements for the two lines. It was hypothesized that the FPCA would be a more precise landmark than the TAL for the determination of the femoral component rotation during TKA.

## Materials and methods

Three hundred and forty-one patients who had undergone TKA owing to osteoarthritis were enrolled in this study. All patients had Kellgren and Lawrence grades 3 and 4 osteoarthritis [16]. Patients who had undergone previous bony surgery or replacement that might have changed the femoral geometry were excluded. Finally, 336 patients (200 female and 136 male) were included. The mean patient age was 68.8 ± 6.4 years (average ± standard deviation). This study was approved by the local ethics committee. MR scans were obtained according to standard protocols for patients with end-stage osteoarthritis a waiting TKA.

**Fig. 1:**
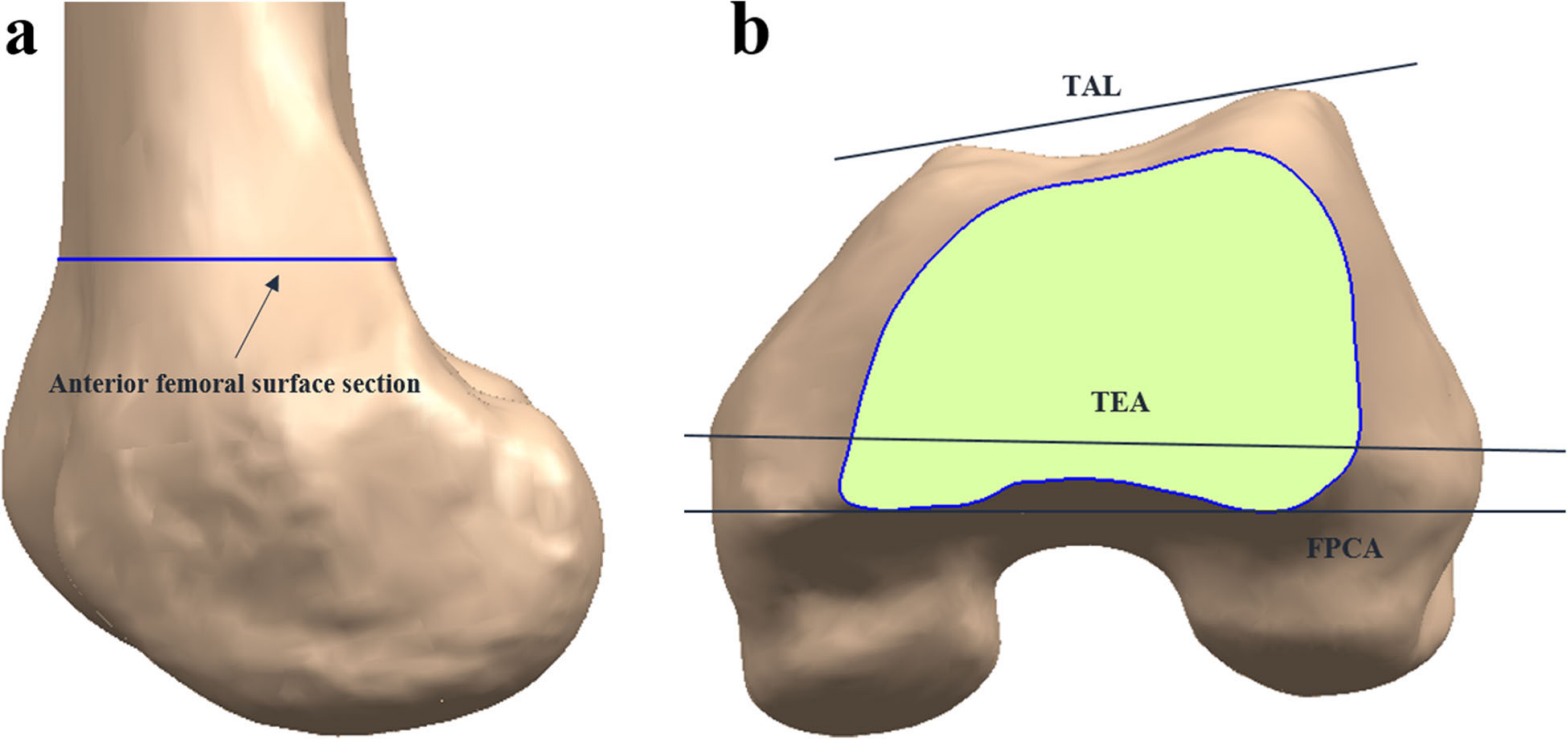
Schematic representations of **a** anterior femoral surface section and **b** TAL, TEA, and FPCA

MR imaging (MRI) was performed using a 1.5-T MR scanner (Achieva 1.5 T; Philips Healthcare, Best, Netherlands). A slice of thickness of 1 mm was used in the sagittal plane for the tibiofemoral knee joint, whereas a slice of thickness of 5 mm was used in the axial plane for the hip and ankle joints. An axial proton density sequence was used to create fat suppression, and a high-resolution setting was used for the spectral pre-saturation inversion recovery sequence (echo time, 25.0 ms; repetition time, 3590.8 ms; acquisition matrix, 512 × 512 pixels; number of excitations, 2.0; field of view, 140 × 140 mm). This method, which is used in patient-specific instances, allowed us to effectively develop three-dimensional (3D) reconstructed models [17]. The MR images were imported into a modeling software (Mimics version 17.0; Materialize, Leuven, Belgium) and segmented to reconstruct the 3D bone and cartilage models of the femur. Then, 3D reconstruction reproducibility analysis was performed, similar to our previous study [18]. Specific anatomical landmarks were identified on the femur from the reconstructed MR images: the hip center, intercondylar notch, two epicondylar points on the medial and lateral sides, and the two most anterior points of the medial and lateral anterior condyles. The femoral mechanical axis was defined as the line connecting the hip center and the intercondylar notch. The anatomical TEA was defined using the medial and lateral epicondyles. The projected TEA line on the plane perpendicular to the mechanical axis was also defined. The anterior femoral surface section was defined as the curve parallel to the plane perpendicular to the mechanical axis (Fig. 1a). The TAL was defined using the most anterior points of the medial and lateral anterior condyles. The FPCA was defined using the previously described anterior femoral surface section (Fig. 1b).

All measurements were performed by an experienced observer who was well-trained in MRI interpretation. To assess the intraobserver and interobserver variability, measurements were repeated on 100 3D MRI reconstructions, from 50 female and 50 male patients, > 1 week after the initial measurements by the same observer and a second observer. The intraobserver and interobserver error (0.89 and 0.92, respectively) were calculated using intra-class correlation.

## Statistical analysis

Statistical analyses were performed using SPSS for Windows (version 12.0; SPSS Inc., Chicago, IL, USA), and Student’s *t* tests were used to determine the significance of coronal alignment and any gender-based differences. The variances between the TAL/TEA and FPCA/TEA angles were compared using *F* tests. Pearson correlation tests were used to evaluate the correlations between the TAL/TEA and FPCA/TEA each angle and the femorotibial angle. The level of significance was set at *p* = 0.05. Power analysis was conducted using G*Power software. The FPCAs of the male and female groups were used to calculate the power values. The alpha value was 0.05, and the power was 0.85.

## Results

No significant differences were observed in the demographic factors between the two groups, including age and body mass index (Table 1). The TAL was internally rotated by 5.2° ± 5.5° relative to the TEA (Table 2).

**Table 1:** Comparison of the age and BMI between Korean males and females

Parameter	Whole patients (*n* = 336)	Female (*n* = 200)	Male (*n* = 136)	*p* value
	Mean ± SD	Mean ± SD	Mean ± SD	
Age	68.8 ± 6.4	68.3 ± 6.5	69.4 ± 6.3	n.s
BMI (kg/m^2^)	23.6 ± 3.2	24.1 ± 3.1	22.9 ± 3.2	n.s

*n.s* non-significant

**Table 2:** Comparison of anthropometric measurements between Korean males and females

Parameter	Whole patients (*n* = 336)	Female (*n* = 200)	Male (*n* = 136)	*p* value
	Mean ± SD (range)	Mean ± SD (range)	Mean ± SD (range)	
TAL/TEA	5.2 ± 5.5 (− 12.8, 20.4)	4.6 ± 4.2 (− 7.6, 16.9)	6.0 ± 6.8 (− 12.8, 20.4)	< 0.05
FPCA/TEA	− 2.6 ± 3.6 (− 10.8, 7.8)	− 3.0 ± 3.5 (− 10.8, 7.8)	− 1.9 ± 3.8 (− 10.0, 7.8)	< 0.05

*n.s* non-significant

The TAL was internally rotated by 4.6° ± 4.2° and 6.0° ± 6.8° relative to the TEA in male and female patients, respectively. However, the FPCA was externally rotated by 2.6° ± 3.6° relative to the TEA (Table 2).

Gender-based differences were also found in the FPCA. The FPCA was externally rotated by 3.0° ± 3.5° and 1.9° ± 3.8° relative to the TEA in the females and males, respectively (Table 2). The *p* value of the variance between the FPCA/TEA and TAL/TEA angles was < 0.05, indicating that the variance in the FPCA/TEA angle was significantly smaller than that in the TAL/TEA (Table 2). The influence of varus or valgus deformities on the reliability of each axis for femoral rotation was also evaluated. The varus-valgus angle (VVA) ranged from − 14.2° (valgus) to 22.9° (varus), with a mean of 7.3° ± 5.8°. The angle between the TAL and the clinical TEA was significantly correlated with VVA (*r* = − 0.22; *p* < 0.01). The angle between the FPCA and the clinical TEA was also significantly correlated with the VVA (*r* = 0.18; *p* < 0.01). Furthermore, both coefficient *r* values showed “weak” correlations.

## Discussion

The most important finding in this study was that the FPCA was better than the TAL for determining the TEA of the distal femur. Because the variance in FPCA/TEA angle was significantly smaller than that in the TAL/TEA angle, the FPCA represented a more appropriate femoral rotational axis than the TAL. Its flat geometry also means that the landmarks on which the axes are based make the FPCA more reproducible than the TAL for both intraoperative and post-operative estimations of the TEA.

Although the importance of proper rotational alignment in TKA is recognized, its determination is controversial compared to that of the axial alignment. The surgical TEA is considered the most commonly used axis for rotational alignment of the femoral component in traditional mechanical alignment when the medial and lateral epicondyles are visible. However, the sulcus of the medial epicondyle and the peak point of the lateral epicondyle are difficult to identify during surgery because they are covered by soft tissues [19, 20], thereby limiting the usefulness of this axis.

The posterior condylar axis and anteroposterior (AP) axes are directly visible and, thus, easier to use under such conditions. Arima et al. [20] have suggested that the AP axis is a reliable landmark that can be used in valgus knees. The AP axis can be constructed on the normal anatomy of the trochlear groove and the intercondylar notch of the distal femur. In knees with arthritis, it is difficult to define the AP axis because of trochlear wear and/or intercondylar osteophytes. These significant arthritic distortions may decrease the reliability of the AP axis.

Researchers have recently suggested two new reference axes in the anterior femur, the TAL/TEA and the femoral anterior tangent line (FAT)/TEA, as alternatives when the conventional reference axes are distorted. Ji et al. determined the reliabilities of these reference axes for femoral component rotation in female patients undergoing TKA by comparing the trochlear and femoral anterior tangent lines [15]. They showed that the TAL/TEA had a significantly smaller variance than the FAT/TEA, demonstrating a more consistent distribution [15]. Matziolis et al. determined the relationship between the anterior and posterior cortical bones in relation to TEA [11] and showed that the posterior cortical bone was a more consistent landmark for intraoperative and postoperative approximations of the TEA than the cortical bone [11].

The TAL showed lesser variance than the FAT in the anterior femoral cortex [15]. Moreover, the posterior cortex of the distal femur appears to be regular, without relevant curvature, showing less variance relative to the TEA [11]. Therefore, TAL and FPCA were investigated, as they showed the least variance, to determine a corresponding femoral component rotational axis. The TAL was internally rotated by 8.1° relative to the FPCA. To the best of our knowledge, this study is the first study to compare the FPCA and the TAL. In a previous study, the TAL was internally rotated by 7.3° ± 1.8° relative to the TEA in healthy knee joints, approximately 2.1° more than that observed in our study [14]. However, in another study, the TAL was internally rotated 5.6° ± 2.3° [13], comparable to our results. In a recent study, the TAL was internally rotated by 6.1° ± 2.5° relative to the TEA, consistent with our findings (5.2° ± 5.5°). Ji et al. showed that the TAL was closely correlated with TEA, and they established an alternative to direct visualization of the TEA [15]. However, our results showed that the TAL has a greater variance in relation to the TEA compared to the FPCA.

Because the posterior cortical bone can always be visualized, even with metal artifacts, it can serve as a control for femoral component rotation in all cases. In addition, the FPCA and the TAL were weakly correlated with varus or valgus deformities of the osteoarthritic knee because these two axes avoid using the distorted condylar and cartilage anatomies to determine rotational alignments of the femoral component. Therefore, the FPCA can be used to determine the intraoperative femoral component rotation in the case of extensive bone defects. In the revision surgery, the TEA is difficult to be defined by PCA because of the posterior bone cutting. The WSL and TAL are also difficult to be defined because of the distal and anterior bone cutting. The FPCA could be the reliable alternative to define the TEA in revision surgery.

Further studies evaluating the femoral component position relative to the FPCA are needed to provide evidence of the biomechanical and clinical significance of this new landmark. However, the measurements of any reliable reference axis, including the FPCA, inevitably vary to some extent between individuals. The composite measurements of multiple axes, TEA, AP axis, or FAT, should provide more appropriate femoral component rotational alignments during TKA. However, access to the FPCA during surgery is difficult because it is measured at the bone behind the capsule. The FPCA can be used in the planning of patient-specific instruments. Because planning is conducted in the 3D bone model, we can access the posterior bone behind the capsule.

This study has some limitations. First, MRI was used to construct the 3D representation of the distal femur, which could have led to errors in the computation model. Nevertheless, MRI allowed us to reconstruct soft tissues such as the articular cartilage, and the inaccuracy in 3D reconstruction was reduced using a previously described protocol [21]. Second, our population lacked ethnic diversity, and the results might differ for other populations. Third, this study did not examine outcome-related alignments, but it defined the anatomy in patients undergoing TKA. Further, we performed the measurements in osteoarthritic knees; therefore, our findings might not be applicable for normal knees. However, our results are useful for surgeons performing TKA.

## Conclusions

Accurate rotational positioning of the femoral component in TKA is extremely important for implant function and longevity. Although there are various landmarks for determining femoral rotational alignment, a single axis is not always sufficient to precisely determine the rotation owing to anatomical variations, poor reproducibility of the axis, and poor visibility of the landmark during surgery. In addition, bone loss observed during revision surgery may further hinder the determination of rotational alignment. The posterior cortical axis is a more consistent landmark than the trochlear anterior line for intraoperative and postoperative approximations of the TEA. These findings demonstrate the usefulness of the posterior cortical axis for determining the rotational alignment of the femoral component before and during TKA.

## Data Availability

The data are available from the corresponding author upon reasonable request.

## Abbreviations

TKA: Total knee arthroplasty
PCA: Posterior cortical axis
TAL: Trochlear anterior line
TEA: Transepicondylar axis
FPCA: Femoral posterior cortical axis
CT: Computed tomography
MR: Magnetic resonance
MRI: Magnetic resonance imaging
VVA: Varus-valgus angle
AP: Anteroposterior
